# Classical simulations of noisy variational quantum circuits

**DOI:** 10.1038/s41534-024-00955-1

**Published:** 2025-05-22

**Authors:** Enrico Fontana, Manuel S. Rudolph, Ross Duncan, Ivan Rungger, Cristina Cîrstoiu

**Affiliations:** 1 Department of Computer and Information Sciences, University of Strathclyde, Glasgow, UK; 2 Quantinuum, Cambridge, UK; 3 National Physical Laboratory, Teddington, UK; 4 Institute of Physics, Ecole Polytechnique Fédérale de Lausanne (EPFL), Lausanne, Switzerland

**Keywords:** Quantum information, Qubits

## Abstract

Noise detrimentally affects quantum computations so that they not only become less accurate but also easier to simulate classically as systems scale up. We construct a classical simulation algorithm, lowesa (low weight efficient simulation algorithm), for estimating expectation values of noisy parameterised quantum circuits with a fixed observable. It combines previous results on spectral analysis of parameterised circuits with Pauli back-propagation and recent ideas for simulations of noisy random circuits. We show, under some conditions on the circuits and mild assumptions on noise, that lowesa gives an efficient, polynomial algorithm in the number of qubits (and depth), with approximation error that vanishes exponentially in the physical error rate and a controllable cutoff parameter. This is valid for any expectation value that may be efficiently evaluated on a quantum computer. We discuss the practical limitations of the method for circuit classes with correlated parameters and its scaling with decreasing error rates.

## Introduction

Quantum hardware has rapidly progressed to enable experiments that reach the barrier where computations become increasingly challenging to simulate with (high performance) classical computing systems^1–5^. Quantum advantage demonstrations recently stimulated substantial advances on classical algorithms for random circuit sampling^6,7^, particularly on approximate tensor networks^8–10^.

In the current quest for applications with suitable implementations on noisy quantum hardware^11^, much recent attention has been devoted to parameterised quantum circuits (PQCs). Variational quantum algorithms (VQAs) combine such a controllable quantum routine with classical optimisation to minimise a cost function that encodes the problem of interest. While they are often considered to have an intrinsic noise-resilience, recent studies have shown that accumulation of errors^12^ leads to phenomena like noise-induced barren plateaus^13^, which hinder and potentially prohibit optimisation^14^. Furthermore, frameworks^15,16^ comparing classical algorithms with noisy VQAs by use of entropic quantities concluded that the circuit depth must have a bound that scales inversely with the physical gate error rate. Beyond this regime, classical methods certifiably outperform the noisy quantum computation^17^. Other classical simulations that target noisy VQAs include decision diagrams^18^ and tensor networks^19,20^. However, these approaches tend to be heuristic and do not necessarily provide rigorous trade-offs between complexity, approximation error, and physical noise.

Here we present lowesa, an efficient classical algorithm for simulating expectation values of PQCs affected by Pauli noise, for fixed generic observables. We combine ideas from simulating noisy random circuit sampling^7^ with spectral decompositions of parameterised noisy quantum circuits^21^. Several applications, particularly in quantum machine learning^22–25^ have used the fact that cost functions for VQAs decompose into (finite) Fourier series in the variational parameters. Our previous work^21,26^ also shows that the effect of noise on the circuit produces Fourier coefficients that are contracted by a factor that decays exponentially with the Hamming weight of the frequency vector ***ω***. The algorithm we propose here produces an approximation to the noisy cost function that consists only of those Fourier modes with frequencies below a fixed cutoff value ∣***ω***∣ ≤ *ℓ*. For Pauli observables, we show the time complexity of lowesa is *O*(*n*^2^*m*2^*ℓ*^) for a *specific class* of circuits on *n* qubits consisting only of *m* independently parameterised non-Clifford gates and any number of Clifford gates. The root mean squared approximation error is shown to decay exponentially with *ℓ* and the physical gate error rate *p*, under mild assumptions on the noise. Equivalently, our algorithm takes $O({n}^{2}m{(\frac{1}{\epsilon })}^{1/p})$) time to produce a function that approximates the noisy cost function within a fixed additive error *ϵ* (averaged over the entire parameter space). Notably, in the noise-less setting, simulating a circuit with *m* non-Clifford gates, as considered here, requires $O(\exp (m))$ time^27^. Improved sub-exponential algorithms for estimation of expectation values assume constant depth and planar architectures^28^. By contrast, not only can we get a linear scaling in *m* in the noisy setting but also, we recover an approximation of the entire cost function landscape rather than a single observable expectation value for a circuit with fixed parameters. The algorithm’s efficiency does not depend on the locality of the observable. For non-Pauli observables, the runtime incurs an additional factor but the algorithm remains efficient whenever the expectation value can also be efficiently evaluated on a quantum computer.

It is important to emphasize that while lowesa is asympotically efficient in qubit number, the exponent scaling with 1/*p* can limit the practical runtime. We leave for further research to investigate the extent to which the algorithm is computationally tractable for finite system sizes and low error rates of *p* ≈ 10^−2^ − 10^−3^ attained by current devices^5^. On the other hand, approximate MPS-based tensor network simulation methods^19^ can also deal with (shallow) noisy circuits of large sizes up to hundreds of qubits, with approximation error that increases significantly with the gate fidelity. However, the complexity has exponential scaling with depth and more intricate circuit topologies beyond 1D. Furthermore, the relation between tensor truncation error and noise becomes difficult to quantify mathematically. It remains an interesting open question if our algorithm can be combined with such tensor network methods to improve the systems sizes accessible via (noisy) classical simulations.

The classical simulation approach presented here does not rely on the specific classical optimisation loop in VQAs, and therefore can be adapted to any algorithm that involves a class of noisy circuits with a fixed structure and observable and independently parameterised gates. For example, certain noisy implementations of quantum signal processing^29,30^ might fall under this.

Finally, our algorithm quantitatively reinforces the idea that gate fidelities of quantum devices need to decrease in order to access regimes beyond classical simulation methods^20^. Increasing the number of qubits for fixed error rates is unlikely to be sufficient as several noisy classical simulation algorithms exhibit polynomial scaling with qubit number, a recurrent feature that also appears in the case of noisy random circuit sampling^6,7^ and tensor network methods^19^.

## Results

### Parameterised quantum circuits

A PQC on *n* qubits is defined as a sequence of *m* unitary gates, each parameterised by a component of a parameter vector ***θ***. Here, we consider the case where the gates are alternating layers of Clifford operations *C*_*i*_ and Pauli rotations ${R}_{i}({\theta }_{i})={e}^{-i{\theta }_{i}/2{P}_{i}}$ where *P*_*i*_ is a multi-qubit Pauli operator. The overall unitary is

1 $$U({\boldsymbol{\theta }})=\left(\mathop{\prod }\limits_{i=1}^{m}{C}_{i}{R}_{i}({\theta }_{i})\right){C}_{0}.$$

The parameters ***θ*** ∈ [0, 2*π*]^*m*^ can therefore be equivalently described as rotation angles. This specific form is operationally relevant as it is featured in many common near-term algorithms^31–33^ and proposals for fault-tolerant architectures^34,35^, and since Clifford unitaries and Pauli rotations form a universal gate set, any PQC can be cast in this way (up to fixing a subset of the parameters).

Typical VQAs involve initialising the quantum computer in $\left\vert {\boldsymbol{0}}\right\rangle ={\left\vert 0\right\rangle }^{\otimes n}$, applying the PQC, and measuring an observable to obtain a cost function. We denote the set of single-qubit Pauli operators by ${\mathbb{P}}=\{I,X,Y,Z\}$ and the expectation value for a specific *n*-qubit Pauli operator $P\in {{\mathbb{P}}}^{\otimes n}$ by

2 $$f({\boldsymbol{\theta }}):= {\rm{tr}}(P\,{{\mathcal{U}}}_{{\boldsymbol{\theta }}}[\left\vert {\boldsymbol{0}}\right\rangle \left\langle {\boldsymbol{0}}\right\vert ]),$$

where the unitary channel is ${{\mathcal{U}}}_{{\boldsymbol{\theta }}}[\cdot ]:= U({\boldsymbol{\theta }})[\cdot ]{U}^{\dagger }({\boldsymbol{\theta }})$. The expectation value of a general observable may be linearly decomposed into expectations of Pauli observables.

### Modelling noisy operations

We are interested in the classical simulatability of VQAs affected by noise, and we model the noisy PQC using general Pauli channels, which are probabilistic mixtures of unitary *n*-qubit Pauli operator evolutions. For a single qubit, a general Pauli channel is given by

3 $$\begin{array}{ll}{{\mathcal{N}}}_{Pauli}({p}_{X},{p}_{Y},{p}_{Z})[\rho ]=(1-{p}_{X}-{p}_{Y}-{p}_{Z})\rho \\\qquad\qquad\qquad\qquad\qquad +{p}_{X}X\rho X+{p}_{Y}Y\rho Y+{p}_{Z}Z\rho Z.\end{array}$$

These are often used to model local decoherent processes in quantum hardware. The dephasing channel ${{\mathcal{N}}}_{Pauli}(0,0,p)$ is a particular example which models interactions between a qubit and the external environment. The best-fit noise parameters {*p*_*X*_, *p*_*Y*_, *p*_*Z*_} for each qubit can be estimated experimentally via procedures like cycle benchmarking^36^.

The noisy circuit model we consider takes the form

4 $${\tilde{{\mathcal{U}}}}_{{\boldsymbol{\theta }}}=\left({\bigcirc}_{i = 1}^{m}\,{{\mathcal{C}}}_{i}\,{\circ}\, {\tilde{{\mathcal{R}}}}_{i}({\theta }_{i})\right)\,{\circ}\, {{\mathcal{C}}}_{0}.$$

The resulting noisy cost function is labelled $\tilde{f}({\boldsymbol{\theta }})$. The symbol “∘” refers to concatenation of maps and “${\bigcirc}_{i = 1}^{m}$” to repeated concatenation. Each noisy gate is given by the target unitary followed by a Pauli channel acting on the subset of qubits *Q*_*i*_ where *P*_*i*_ acts nontrivially. Specifically, we have

5 $${\tilde{{\mathcal{R}}}}_{i}({\theta }_{i})=\bigotimes _{q\in {Q}_{i}}{{\mathcal{N}}}_{Pauli}^{(q)}\,{\circ}\, {{\mathcal{R}}}_{i}({\theta }_{i}).$$

We will later consider more general noise model where the Clifford operations also incur noise: ${\tilde{{\mathcal{C}}}}_{i}={{\mathcal{C}}}_{i}\,{\circ}\, {\mathcal{M}}$, where ${\mathcal{M}}$ are multi-qubit Pauli channels, and where noise is allowed to vary across the circuit.

Finally, let us define $p={\min }_{\sigma = X,Y,Z}{p}_{\sigma }.$ Generally real devices will have a symmetric depolarising component to every operation so we can assume *p* > 0 holds^13^.

### Pauli transfer matrices and simulation algorithms

When studying generic quantum operations it can often be useful to use the *Pauli transfer matrix* (PTM) formalism^37^. Let us briefly review it. In the PTM formalism, one takes the view of the normalised Pauli basis $\widehat{{\mathbb{P}}}=\frac{1}{\sqrt{2}}\{I,X,Y,Z\}$, where a normalised Pauli operator ${\hat{P}}_{i}\in {\hat{{\mathbb{P}}}}^{\otimes n}$ is a basis vector $\left.\left\vert {P}_{i}\right\rangle \right\rangle$ in the space ${{\mathbb{R}}}^{{4}^{n}}$. The normalisation ensures that $\left\langle \left\langle {P}_{i}| {P}_{j}\right\rangle \right\rangle ={\rm{tr}}({\hat{P}}_{i}{\hat{P}}_{j})={\delta }_{ij}$. Quantum states can be written in this basis as $\left.\left\vert \rho \right\rangle \right\rangle$,

6 $${[\left.\left\vert \rho \right\rangle \right\rangle ]}_{i}={\rm{tr}}(\rho {\hat{P}}_{i}),$$

extending the identification of a one-qubit density matrix with its Bloch vector to higher dimensions. For instance, in this basis we represent the density matrix $\left\vert 0\right\rangle \left\langle 0\right\vert$ as $\left.\left\vert 0\right\rangle \right\rangle =[1/\sqrt{2},0,0,1/\sqrt{2}]$. Then, a quantum channel ${\mathcal{E}}$ is a matrix (the PTM) ${\bf{E}}\in {{\mathbb{R}}}^{{4}^{n}\times {4}^{n}}$,

7 $${[{\bf{E}}]}_{ij}=\left\langle \left\langle {P}_{i}| {\bf{E}}| {P}_{j}\right\rangle \right\rangle ={\rm{tr}}({\hat{P}}_{i}{\mathcal{E}}[{\hat{P}}_{j}]),$$

and therefore expectation values of Pauli operators are written as $\left\langle \left\langle {P}_{i}| {\bf{E}}| \rho \right\rangle \right\rangle ={\rm{tr}}({\hat{P}}_{i}{\mathcal{E}}[\rho ])$. Composition of quantum channels becomes matrix multiplication.

The PTM formalism can be used to calculate expectation values in the *Heisenberg picture* via Pauli back-propagation, where the quantum channels are seen as acting on the measurement operator instead of the state^38^. In PTM form this adjoint operation corresponds to simply taking the transpose of the expectation value

8 $$\left\langle \left\langle P| {\bf{E}}| \rho \right\rangle \right\rangle =\left\langle \left\langle \rho | {{\bf{E}}}^{{\mathsf{T}}}| P\right\rangle \right\rangle ,$$

which is possible for any ${\mathcal{E}}$. This perspective provides an efficient approach to classically computing expectation values. Take an *n*-qubit channel ${\mathcal{E}}$ and assume it can be decomposed as a sum of *N* Clifford unitary channels ${{\mathcal{E}}}_{i}$ via ${\mathcal{E}}=\mathop{\sum }\nolimits_{i = 1}^{N}{c}_{i}{{\mathcal{E}}}_{i}$, ∑_*i*_*c*_*i*_ = 1. Also consider a stabiliser state^38^*ρ* such that the expectation value with any Pauli operator can be evaluated efficiently. Then, given a Pauli *P*, the expectation value $\left\langle \left\langle P| {\bf{E}}| \rho \right\rangle \right\rangle$ can be expanded as a sum of *N* terms $\left\langle \left\langle P| {{\bf{E}}}_{i}| \rho \right\rangle \right\rangle$. As Clifford unitaries are generalised permutation matrices in the PTM representation we get $\left\langle \left\langle \rho | {{\bf{E}}}_{i}^{{\mathsf{T}}}| P\right\rangle \right\rangle =\left\langle \left\langle \rho | {P}_{i}^{{\prime} }\right\rangle \right\rangle$ (up to a phase), for some other Pauli operator ${P}_{i}^{{\prime} }$. When ${{\mathcal{E}}}_{i}$ is an *n*-qubit Clifford unitary then it can be synthesised into at most *O*(*n*^2^/*log*(*n*)) gates^39^ and the change of Pauli frame from *P* to ${P}_{i}^{{\prime} }$ can be efficiently computed in *O*(*n*^2^)^38,40^. Finally, since *ρ* is assumed a stabiliser state, the expectation value $\left\langle \left\langle \rho | {{\bf{E}}}_{i}^{{\mathsf{T}}}| P\right\rangle \right\rangle$ can be efficiently computed in *O*(*n*^2^). This gives an efficient classical algorithm to compute expectation values when *N* ~ poly(*n*).

### Prior art

This approach is not new. The decomposition of general channels into sums of stabiliser channels (Cliffords and Pauli measurements) for the purpose of quantum circuit simulation was introduced in ref. ^41^. A similar sum-over-Clifford algorithm for unitary circuits was explored in ref. ^27^. A PTM-based algorithm for both exact and noisy circuit simulation has been proposed in ref. ^42^ from a Schrödinger perspective (state propagation). The work in ref. ^43^ is the closest to the method used here, as it covers the PTM representation in conjunction with a Heisenberg picture simulation method. In addition, it discusses the effect on simulatability of adding symmetric depolarising noise on *z*-rotation gates.

However, something that to our knowledge has not been made explicit before is that the method can be generalised beyond decompositions into Clifford unitaries (or near-Clifford unitaries^27^) and Pauli measurement channels. Indeed, here we will consider general processes ${{\mathcal{E}}}_{i}$ for which the expectation value $\left\langle \left\langle P| {{\bf{E}}}_{i}| \rho \right\rangle \right\rangle$ can be evaluated efficiently. Notably, the processes ${{\mathcal{E}}}_{i}$ need not even be valid quantum channels (or completely positive trace preserving maps), we only require that its PTM representation is sufficiently sparse. This occurs when the adjoint channel ${{\mathcal{E}}}_{i}^{\dagger }$ maps every Pauli operator into a combination of small, *O*(poly(*n*)), number of Pauli operators. This echoes remarks in ref. ^44^, although that work is in the Schrödinger picture. In our case, the ${{\mathcal{E}}}_{i}$ will correspond to compositions of Clifford unitaries and processes that map every Pauli operator to a single (possibly distinct) Pauli operator or to zero.

### Strategy

We first show how the noisy variational circuits considered in Eq. (1) admit a linear decomposition into processes that are amenable to the classical simulation outlined in “Pauli transfer matrices and simulation algorithms ”. To that aim, it turns out that a decomposition into Fourier series of the noisy channel ${\tilde{{\mathcal{U}}}}_{\theta }$, and therefore noisy cost function, results in processes that map a Pauli operator into multiple Pauli operators, and thus their composition may lead to an exponential accumulation of terms. However, a different choice of basis involving trigonometric polynomials remedies this to produce a decomposition for which the dominant coefficients in the expansion can be efficiently computed.

We now assume that all rotations are single qubit *z*-rotations. This is purely to make the exposition easier to follow, the theorems will be valid for any Pauli rotation. Let **R**_*z*_(*θ*) be the PTM of ${{\mathcal{R}}}_{z}(\theta )$ and let **N** be the PTM of the Pauli noise channel ${{\mathcal{N}}}_{Pauli}$, **N** = diag(1, *q*_*X*_, *q*_*Y*_, *q*_*Z*_). The eigenvalues of the Pauli channel are related to the error probabilities as *q*_*X*_ = 1 − 2(*p*_*Z*_ + *p*_*Y*_), *q*_*Y*_ = 1 − 2(*p*_*Z*_ + *p*_*X*_), *q*_*Z*_ = 1 − 2(*p*_*X*_ + *p*_*Y*_). Then, the noisy channel ${\tilde{{\mathcal{R}}}}_{z}(\theta )={{\mathcal{N}}}_{Pauli}\,{\circ}\, {{\mathcal{R}}}_{z}(\theta )$ has, with respect to the orthonormal basis $\{\left.\left\vert I\right\rangle \right\rangle ,\left.\left\vert X\right\rangle \right\rangle ,\left.\left\vert Y\right\rangle \right\rangle ,\left.\left\vert Z\right\rangle \right\rangle \}$, the PTM

9 $${\bf{N}}\cdot {\bf{R}}=\left(\begin{array}{cccc}1&0&0&0\\ 0&{q}_{X}\cos \theta &-{q}_{X}\sin \theta &0\\ 0&{q}_{Y}\sin \theta &{q}_{Y}\cos \theta &0\\ 0&0&0&{q}_{Z}\end{array}\right).$$

Denote the projectors by ${\Pi }_{0}=\left.\left\vert I\right\rangle \right\rangle \left\langle \left\langle I\right\vert \right.+\left.\left\vert Z\right\rangle \right\rangle \left\langle \left\langle Z\right\vert \right.$, ${\Pi }_{X}=\left.\left\vert X\right\rangle \right\rangle \left\langle \left\langle X\right\vert \right.$ and ${\Pi }_{Y}=\left.\left\vert Y\right\rangle \right\rangle \left\langle \left\langle Y\right\vert \right.$. Then we can define new quantum processes $\{{{\mathcal{D}}}_{0},{{\mathcal{D}}}_{1},{{\mathcal{D}}}_{-1}\}$ to be used in the simulation algorithm via their PTM representation **D**_**0**_ = Π_0_**NR**Π_0_, **D**_**1**_ = Π_*X*_**NR**Π_*X*_ + Π_*Y*_**NR**Π_*Y*_ and **D**_**-1**_ = Π_*X*_**NR**Π_*Y*_ + Π_*Y*_**NR**Π_*X*_ such that

10 $${\bf{N}}\cdot {\bf{R}}={{\bf{D}}}_{{\bf{0}}}+\cos \theta \,{{\bf{D}}}_{{\bf{1}}}+\sin \theta \,{{\bf{D}}}_{-{\bf{1}}}.$$

Expanding out these processes, we see that each of them maps any single Pauli operator into at most another single Pauli operator (up to a scaling),

11 $${{\bf{D}}}_{0}=\left(\begin{array}{cccc}1&0&0&0\\ 0&0&0&0\\ 0&0&0&0\\ 0&0&0&{q}_{Z}\end{array}\right),{{\bf{D}}}_{1}=\left(\begin{array}{cccc}0&0&0&0\\ 0&{q}_{X}&0&0\\ 0&0&{q}_{Y}&0\\ 0&0&0&0\end{array}\right),$$

12 $${{\bf{D}}}_{-1}=\left(\begin{array}{cccc}0&0&0&0\\ 0&0&-{q}_{X}&0\\ 0&{q}_{Y}&0&0\\ 0&0&0&0\end{array}\right).$$

This decomposition allows us to expand the noisy circuits in terms of a multivariate trigonometric basis, which is a more convenient choice for the classical simulation. Consider ${\Phi }_{{\boldsymbol{\omega }}}({\boldsymbol{\theta }}):= \mathop{\prod }\nolimits_{i = 1}^{m}{\phi }_{{\omega }_{i}}({\theta }_{i})$ where ${\phi }_{0}(\theta )=1,\,{\phi }_{1}(\theta )=\cos (\theta ),\,{\phi }_{-1}(\theta )=\sin (\theta )$ are *trigonometric monomials* that encode the ***θ*** dependence. Then, the *noisy* variational circuits admit the decomposition

13 $${\tilde{{\mathcal{U}}}}_{\theta }=\sum _{{\boldsymbol{\omega }}\in {\{0,\pm 1\}}^{m}}{\Phi }_{{\boldsymbol{\omega }}}({\boldsymbol{\theta }}){{\mathcal{D}}}_{{\boldsymbol{\omega }}},$$

where each process ${{\mathcal{D}}}_{{\boldsymbol{\omega }}}$ is labelled by a frequency vector ***ω*** ∈ [0, ±1]^*m*^ and given by

14 $${{\mathcal{D}}}_{{\boldsymbol{\omega }}}:= \left({\bigcirc}_{i}\,{\tilde{{\mathcal{C}}}}_{i}\,{\circ}\, {{\mathcal{D}}}_{{\omega }_{i}}\right)\,{\circ}\, {{\mathcal{C}}}_{0}.$$

In keeping with previous work^26^ we call these channels *process modes*. Crucially, each process mode maps one Pauli operator onto another Pauli operator, as can be seen from the PTMs above and the defining property of Clifford operations.

Overall, this new decomposition yields the following Fourier series representation for the cost function in Equation (2)

15 $$\tilde{f}({\boldsymbol{\theta }})=\sum _{{\boldsymbol{\omega }}}{d}_{{\boldsymbol{\omega }}}{\Phi }_{{\boldsymbol{\omega }}}({\boldsymbol{\theta }}).$$

The *Fourier coefficients* are given by

16 $${d}_{{\boldsymbol{\omega }}}:= {\rm{tr}}(P{{\mathcal{D}}}_{{\boldsymbol{\omega }}}[\left\vert 0\right\rangle \left\langle 0\right\vert ])=\sqrt{{2}^{n}}\left\langle \left\langle P| {{\bf{D}}}_{{\boldsymbol{\omega }}}| 0\right\rangle \right\rangle ,$$

where the factor $\sqrt{{2}^{n}}$ is necessary since we have defined *f*(***θ***) as the expectation value of an unnormalised Pauli operator.

Note that in the above, the Clifford unitaries ${{\mathcal{C}}}_{i}$ were noise-free and the parameterised rotation gates carried a time-independent Pauli noise. A similar decomposition arises when we consider the general Pauli noise model for ${\tilde{{\mathcal{C}}}}_{i}={{\mathcal{M}}}_{i}\,{\circ}\; {{\mathcal{C}}}_{i}$. In this case, we denote the resulting process modes by ${{\mathcal{D}}}_{{\boldsymbol{\omega }}}^{{\prime} }:= ({\bigcirc}_{i}\,{{\mathcal{M}}}_{i}\,{\circ}\, {{\mathcal{C}}}_{i}\,{\circ}\, {{\mathcal{D}}}_{{\omega }_{i}})\,{\circ}\, {{\mathcal{M}}}_{0}\,{\circ}\, {{\mathcal{C}}}_{0}$ and the corresponding coefficients by ${d}_{{\boldsymbol{\omega }}}^{{\prime} }=\sqrt{{2}^{n}}\left\langle \left\langle P| {{\bf{D}}}_{{\boldsymbol{\omega }}}^{{\prime} }| 0\right\rangle \right\rangle$. We first describe and analyse the proposed classical algorithm for the simpler noise model that only affects the parameterised gates. This is purely to make the exposition easier to follow. The same principle works in the general case (see “ General Pauli noise models”). Furthermore, the analysis extends to time-dependent Pauli errors. The noise models considered here also include the local depolarising channels that have been previously used in classical algorithms for noisy random circuit sampling^7^. Both in our case and in previous work there is an implicit assumption that the Pauli error probabilities for each gate are known a-priori.

### The LOWESA simulation algorithm

We are now in a position to state the simulation algorithm, which shares similar features to the algorithm in ref. ^6^, but applied to the task of estimating expectation values and to a different family of circuits. We name it lowesa for low weight efficient simulation algorithm (pronounced “low-EE-sa”).

Given a cutoff parameter *ℓ*, lowesa returns a *function*$\tilde{g}$ approximating the noisy cost function $\tilde{f}$ constructed from all the low Hamming weight ∣***ω***∣ ≔ ∥***ω***∥_1_ ≤ *ℓ* terms. This function is expressed as a trigonometric series and can therefore be used to evaluate the cost estimate for any parameter vector ***θ*** using

17 $$\tilde{g}({\boldsymbol{\theta }})=\sum _{| {\boldsymbol{\omega }}| \le \ell }{d}_{{\boldsymbol{\omega }}}{\Phi }_{{\boldsymbol{\omega }}}({\boldsymbol{\theta }})$$

with low computational effort. As the algorithm produces all ${\{{d}_{{\boldsymbol{\omega }}}\}}_{| {\boldsymbol{\omega }}| \le l}$, the function evaluation is independent of qubit number and depth.

lowesa involves the following steps:

#### Algorithm 1

[**LOWESA**] Simulating cost functions of noisy VQAs with uncorrelated angles

**Input:** Quantum process given by Eq. (4) defined by process modes as in Eq. (14); measurement Pauli operator *P*; cutoff parameter *ℓ*.

**Output:** $\tilde{g}({\boldsymbol{\theta }})$, an approximation of $\tilde{f}({\boldsymbol{\theta }})$.

 1: **procedure** lowesa

 2:   $$\tilde{g}({\boldsymbol{\theta }})\leftarrow 0$$

 3:   **run** Branch(*P*, (), *m*) recursively to yield ${d}_{{\boldsymbol{\omega }}}=\sqrt{{2}^{n}}\left\langle \left\langle 0| {{\bf{D}}}_{{\boldsymbol{\omega }}}^{{\mathsf{T}}}| P\right\rangle \right\rangle \,\forall \,| {\boldsymbol{\omega }}| \le \ell$.

 4:   **for all** non-zero *d*_***ω***_ **do**

 5:     $$\tilde{g}({\boldsymbol{\theta }})\leftarrow \tilde{g}({\boldsymbol{\theta }})+{d}_{{\boldsymbol{\omega }}}{\Phi }_{{\boldsymbol{\omega }}}({\boldsymbol{\theta }})$$

 6:   **end for**

 7:   **return** $$\tilde{g}({\boldsymbol{\theta }})$$

 8: **end procedure**

**Subroutine:** Calculate *d*_***ω***_ ∀ ∣***ω***∣≤*ℓ* via recursion.

 1: **procedure** Branch(*Q*, ***ω***, *i*)

 2:   $$Q\leftarrow {{\mathcal{C}}}_{i}^{\dagger }(Q)$$

 3:   **if** *i* > 0 **then**

 4:     **if** [*Q*, *P*_*i*_] = 0 **then**

 5:      Branch($${{\mathcal{D}}}_{0}^{i\dagger }(Q)$$, append(***ω*** ← 0), *i* − 1)

 6:     **else if** ∣***ω***∣ < *ℓ* **then**

 7:      Branch($${{\mathcal{D}}}_{1}^{i\dagger }(Q)$$, append(***ω*** ← 1), *i* − 1)

 8:      Branch($${{\mathcal{D}}}_{-1}^{i\dagger }(Q)$$, append(***ω*** ← − 1), *i* − 1)

 9:     **else**

10:      **break**

11:     **end if**

12:   **end if**

13:   **yield** $${d}_{{\boldsymbol{\omega }}}=\sqrt{{2}^{n}}\left\langle \left\langle 0| Q\right\rangle \right\rangle $$

14: **end procedure**

We shall now explain how Algorithm 1 works step by step and why it is efficient. Note that, while our exposition here deals with expectation values of a single Pauli operator, the results extend immediately to general observables as explained in “ General measurement operators”.

**Fig. 1:**
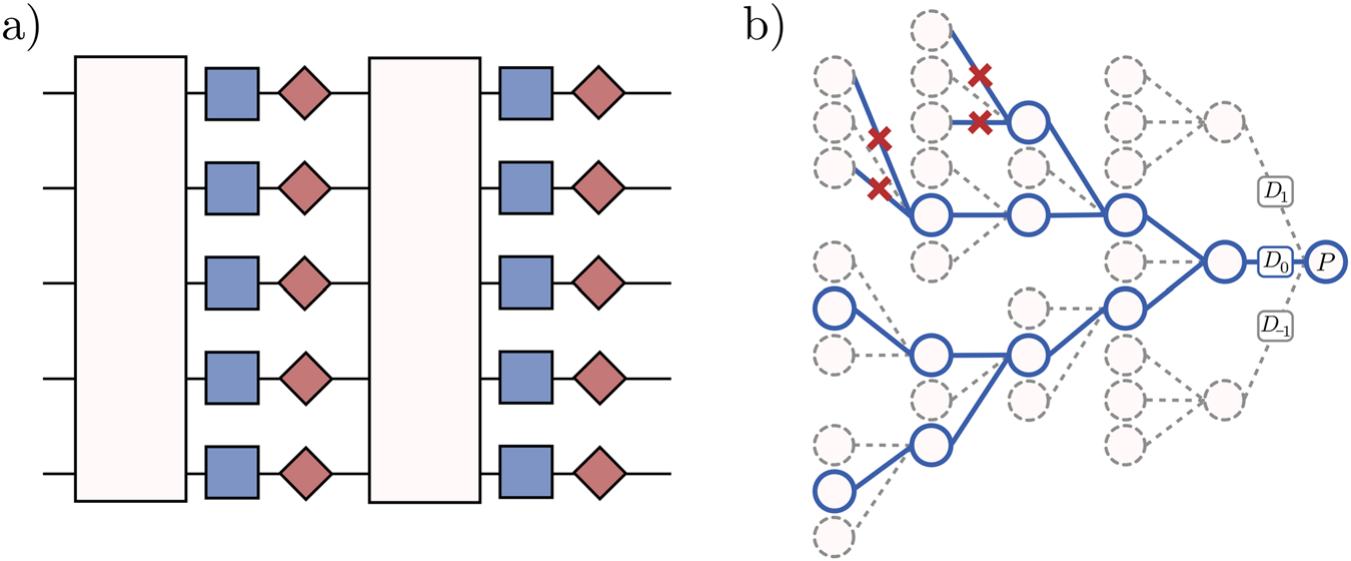
**Circuit model and algorithm flow. a** Schematic of the parameterised quantum circuits that can be simulated by lowesa. The light boxes are arbitrary (noisy) Clifford gates, the blue boxes are parameterised Pauli rotations and the red kites represent Pauli noise channels. **b** Diagrammatic sketch of lowesa as described in Algorithm 1 applied to circuits given by Eq. (1). The Pauli operator *P* is propagated backwards through the circuit where every Clifford gate transforms it into another Pauli, and the decomposition of the parameterised Pauli rotations into process modes *D*_0_, *D*_1_, *D*_−1_ splits the propagation up into paths that may annihilate. A cutoff of *ℓ* = 2 is chosen which artificially annihilates paths that branch into *D*_1_, *D*_−1_ more than 2 times.

Start with the target Pauli measurement operator *P* and propagate in the Heisenberg picture through the circuit. For each Clifford unitary *C*_*i*_, updating the Pauli operator (by conjugation) takes at most *O*(*n*^2^). Each process mode ${{\mathcal{D}}}_{{\omega }_{i}}^{i}$ within a path ${{\mathcal{D}}}_{{\boldsymbol{\omega }}}$ acts with Pauli generator *P*_*i*_. Note that the superscript indicates to which gate it corresponds and the subscript *ω*_*i*_ ∈ {0, ± 1} labels the type of mode. The gate label is necessary since owing to the different generators *P*_*i*_ the process modes may be different, however their effect on an arbitrary Pauli be easily evaluated making classical simulation possible. If the propagated Pauli operator commutes with *P*_*i*_ then only ${{\mathcal{D}}}_{0}^{i}$ leads to a non-zero path, otherwise if it anticommutes either ${{\mathcal{D}}}_{1}^{i}$ or ${{\mathcal{D}}}_{-1}^{i}$ are valid choices. See Fig. 1.

**Fig. 2:**
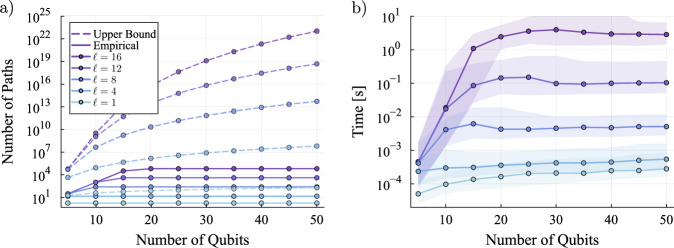
Scaling of lowesa with the number of qubits *n* and cutoff parameter *ℓ.* The circuit structure consists of two parameterised layers of *H* − *Rz*(*θ*_*i*_) − *X* − *H* on each qubit, where the Hadamard and X gates are chosen with 0.5 probability, followed by CNOTs placed on a 2D topology. **a** Upper bound on the number of paths for a given *ℓ*, which equals $$\mathop{\sum }\nolimits_{i = 0}^{\ell }\left(\begin{array}{c}m\\ i\end{array}\right){2}^{i}$$, and the median number of paths empirically explored by lowesa, which is dramatically lower. **b** Wall time to run lowesa with truncation parameter *ℓ* on an average laptop without parallelisation. Each data point represents an average over 1000 different randomised circuits with Pauli Z measurement operators that act on a random subset of qubits. The shading shows the 90% confidence interval. The simulation of the Clifford gates used a look-up table, meaning that the scaling in *n* is entirely due the scaling of *m* with *n*.

As only ${{\mathcal{D}}}_{\pm 1}^{i}$ contribute to the total weight ∣***w***∣ and since we impose ∣***w***∣ ≤ *ℓ*, it suggests a binary tree-like data structure with *ℓ* layers to keep track of the change of Pauli frame and the different branching possibilities. A branch may terminate sooner than if it propagated the Pauli through the entire circuit. The number of branches and therefore valid paths ${{\mathcal{D}}}_{\omega }$ will be at most 2^*ℓ*^. Putting everything together, this reduces the total complexity of evaluating all non-zero *d*_***ω***_ with ∣***ω***∣ ≤ *ℓ* to *O*(*n*^2^*m*2^*ℓ*^) in the worst case. We note that the quadratic scaling in *n* is for general *n*-qubit Clifford unitaries, and can be improved for *k*-local (or sparse) unitaries. In particular, if one fixes the set of Clifford unitaries that are executed within the circuits (for example the set {*X*, *H*, *CNOT*}), one can employ time-memory trade-off tools like look-up tables for each *k*-body Clifford operation and how they act on every *k*-body Pauli operator. In Fig. 2 we illustrate the runtime of lowesa using this technique on a circuit structure that is typically challenging for classical simulators.

To analyse the asymptotic complexity, we need to (1) show that each term *d*_***ω***_ can be efficiently estimated, (2) count the number of non-zero process modes ${{\mathcal{D}}}_{{\boldsymbol{\omega }}}$ with ∣***ω***∣ ≤*ℓ* , and (3) evaluate the accuracy in the approximation $\tilde{g}\approx \tilde{f}$. Condition (1) is satisfied by construction - the choice of trigonometric basis ensures that (the adjoint of) ${{\mathcal{D}}}_{{\boldsymbol{\omega }}}$ maps a Pauli operator to either zero or a (different, scaled) Pauli operator. Each *d*_***ω***_ can be individually estimated in at most *O*(*n*^2^*m*) steps using the Pauli back-propagation method outlined in “ Pauli transfer matrices and simulation algorithms”. For (2), note that while there are $$(\begin{array}{c}m\\ | {\boldsymbol{\omega }}| \end{array})\,{2}^{| {\boldsymbol{\omega }}| }$$ paths with a fixed weight ∣***ω***∣ for a total of at most ${m}^{{\mathcal{O}}(\ell )}$ within the cutoff, many of these will be zero when acted upon the input $\left.\left\vert P\right\rangle \right\rangle$. This is due to the fact that process modes in Equation (11) each annihilate half of the Paulis.

Finally, condition (3) remains to be verified so that lowesa yields an accurate simulation of the noisy cost function. Given a cost function $\tilde{f}$ and its approximation $\tilde{g}$, we define the average *L*^2^-norm error over the space of parameters Θ = [0, 2*π*]^*m*^ or root mean squared error (RMSE)

18 $$\Delta (\tilde{f},\tilde{g}):= {\left(\frac{1}{| \Theta | }{\int}_{\Theta }| \tilde{f}({\boldsymbol{\theta }})-\tilde{g}({\boldsymbol{\theta }}){| }^{2}d{\boldsymbol{\theta }}\right)}^{1/2},$$

where the integration measure is *d****θ*** = *d* *θ*_1_*d* *θ*_2_…*d* *θ*_*m*_ and ∣ *Θ* ∣ = (2*π*)^*m*^ is a normalisation factor so that $\frac{1}{| \Theta | }\int\,d\,{\boldsymbol{\theta }}=1$. In Methods we prove the following result:

#### Theorem 1

Consider a *n*-qubit VQA with a PQC as in Eq. (1) having *m* independently parameterised rotations affected by a single-qubit Pauli noise channel ${{\mathcal{N}}}_{Pauli}({p}_{X},{p}_{Y},{p}_{Z})$ as in Eq. (4). Recall that $p=\mathop{\min}\nolimits_{\sigma = X,Y,Z}{p}_{\sigma }\, > \,0$.

Then, for any weight cutoff $\ell \in {\mathbb{N}}$, lowesa (Algorithm 1) returns an approximation $\tilde{g}$ for the noisy cost function $\tilde{f}$ with RMSE

19 $$\Delta (\tilde{f},\tilde{g})\le {(1-2p)}^{\ell +1}\le {e}^{-2p\ell }$$

and runs in time at most *O*(*n*^2^*m* 2^*ℓ*^).

It follows from Theorem 1 that lowesa is both accurate and efficient, as its runtime scales polynomially with *m* and *n* and the maximum allowed RMSE; however, the scaling with noise probability is considerably worse. For example, suppose we wish to have an error bounded by *ϵ*. Then one would choose $\ell \approx \frac{1}{2p}\log {\epsilon }^{-1}$, giving a runtime $O({\epsilon }^{-\frac{\log 2}{2p}}\,{n}^{2}m)$. While this is asymptotically efficient in the width and depth of the circuit, the dependency on the error rate limits its practicality. Notably the exponent may still be considerably large if the noise is small. When the goal is to simulate the expected outcome of a hardware implementation with a finite number of measurements *N*_*s*_, the error can be chosen like $\epsilon \in {\mathcal{O}}(\frac{1}{\sqrt{{N}_{s}}})$, thus relaxing the precision requirements.

**Fig. 3:**
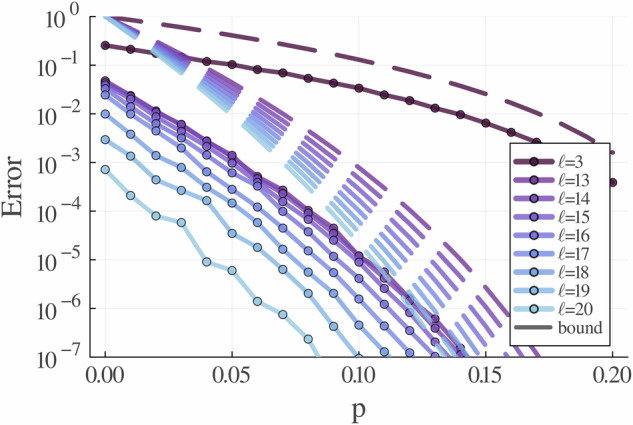
Accuracy benchmark of lowesa compared to the error bounds as predicted in Theorem 1. We show the *L*^2^ error of a single-qubit Pauli Y operator expectation with *ℓ* < *m* = 60 for two layers of a *n* = 10 qubit circuit. The circuit consists of parametrised single-qubit gates *R*_*z*_(***θ***_*i*_) *R*_*x*_(***θ***_*i*+1_) *R*_*z*_(***θ***_*i*+2_) on each qubit followed by CNOT gates in a 2D topology. For this particular circuit, each entangler in the 2D topology was placed with a 0.5 probability. The noise model is symmetric depolarising noise, where the parameters are set *p*_*X*_ = *p*_*Y*_ = *p*_*Z*_ = *p*. Each point is averaged over 1000 random parameterisations of the same circuit to compare to the integral definition of our error bounds. All paths below *ℓ* = 3 and above *ℓ* = 21 annihilate. Consequently, the simulation with *ℓ* = 21 is exact.

In Fig. 3 we illustrate the mean accuracy of the algorithm for an example circuit of the hardware-efficient family. We observe that the error is typically up to two orders of magnitude lower than the bounds, suggesting these are loose and may be improved for the typical case.

### Validity of error measure

The use of RMSE as error measure has limitations, the main one being that the error at any given point is in principle unbounded. However we argue that this limitation is weaker than may appear. Applying Markov’s inequality to our Theorem we have the following probabilistic bound:

#### Corollary 2

For a fixed circuit, choosing the parameters ***θ*** uniformly at random from [0, 2*π*]^*m*^, with probability ≥1 − *δ* the approximation error is bounded by

20 $$| \tilde{f}({\boldsymbol{\theta }})-\tilde{g}({\boldsymbol{\theta }})| \le {e}^{-p\ell }{\delta }^{-1/2}$$

Suppose that we wish to have error bounded by *ϵ* with probability 1 − *δ*. Then the required cutoff is $\ell \approx {p}^{-1}\log ({\epsilon }^{-1}{\delta }^{-1/2})$, giving again a runtime that scales unfavourably with *p*. However for fixed *p* the scaling is logarithmic in both *δ* and *ϵ* meaning that the probability of encountering large deviations can be made arbitrarily small by increasing *ℓ*. This probabilistic formulation has practical relevance as typical VQAs have their parameters initialised uniformly at random^45^ and so it is valid at initialisation; however, this analysis breaks down when considering the error over the whole path of gradient descent, which may lead into a region of high deviation.

### General measurement operators

Up to now we assumed that the measurement operator is Pauli, however in truth most practical algorithms have more complicated measurement operators. Generally, a measurement takes the form

21 $$O=\sum _{{P}_{i}\in {{\mathbb{P}}}^{n}/\{{I}^{n}\}}{c}_{i}{P}_{i}$$

where we can ignore the identity component as it contributes a constant to the cost function. We get the following result, proven in Methods:

#### Theorem 3

With a general measurement operator as in Eq. (21), LOWESA can simulate the noisy cost function with RMSE Δ ≤ *ϵ* and with runtime at most

22 $$O\left({(\parallel {\boldsymbol{c}}{\parallel }_{r}{\epsilon }^{-1})}^{\frac{\log 2}{2p}}{n}^{2}m \right).$$

Assuming $$p\,\ll\, \log\, \sqrt{2}$$, *r* ≈ 1.

The approach consists in separately simulating each Pauli observable composing *O*, allocating to *P*_*i*_ a cutoff budget of ${\ell }_{i}=\frac{1}{2p+\log 2}\log | {c}_{i}| +\,\text{const.}\,$, which gives the minimal error for a given total runtime. The procedure is highly parallelisable meaning that the actual running time can be reduced considerably from these estimates.

Comparing this with the maximum number of shots required by a quantum computer to approximate a composite observable with precision *ϵ*, ${N}_{s}=\parallel {\boldsymbol{c}}{\parallel }_{1}^{2}{\epsilon }^{-2}$^46^, we conclude that when $p\ll\, \log\, \sqrt{2}$ lowesa incurs a cost at most polynomially larger than usual sampling cost. Once again, in practical scenarios the factor of *p*^−1^ will dominate the exponent but this does not invalidate the claim of classical simulatability. Thus we conclude that any expectation value that can be measured efficiently on a quantum computer may be efficiently simulated using our algorithm.

### General Pauli noise models

The result can be extended to cover multi-qubit Pauli noise affecting all gates, not just the parameterised ones. In Methods we prove the more general result:

#### Theorem 4

Consider an *n*-qubit VQA under the noise model

23 $${\tilde{{\mathcal{U}}}}_{{\boldsymbol{\theta }}}=\left({\bigcirc}_{i = 1}^{m}\,{{\mathcal{M}}}_{i}\,{\circ}\, {{\mathcal{C}}}_{i}\,{\circ}\, {{\mathcal{N}}}_{i}\,{\circ}\, {{\mathcal{R}}}_{i}({\theta }_{i})\right)\,{\circ}\, {{\mathcal{M}}}_{0}\,{\circ}\, {{\mathcal{C}}}_{0}$$

where $\{{{\mathcal{M}}}_{i}\}$ are *n*-qubit Pauli channels with layer-dependent noise parameters and every rotation is followed by a local multi-qubit Pauli noise ${{\mathcal{N}}}_{i}{ = \bigotimes }_{j = 1}^{n}{{\mathcal{N}}}_{Pauli}^{(j)}({p}_{X}^{ij},{p}_{Y}^{ij},{p}_{Z}^{ij})$ with ${p}^{{\prime} }=\mathop{\min}\nolimits_{ij\sigma }\{{p}_{\sigma }^{ij}\} > 0$, which depends on both layer and qubit. Then, for any weight cutoff $\ell \in {\mathbb{N}}$, lowesa (Algorithm 1) with modified process modes ${{\mathcal{D}}}_{{\boldsymbol{\omega }}}^{{\prime} }=({\bigcirc}_{i = 1}^{m}{{\mathcal{M}}}_{i}\,{\circ}\, {{\mathcal{C}}}_{i}\,{\circ}\, {{\mathcal{D}}}_{{\omega }_{i}})\,{\circ}\, {{\mathcal{C}}}_{0}\,{\circ}\, {{\mathcal{M}}}_{0}$ and coefficients ${d}_{{\boldsymbol{\omega }}}^{{\prime} }=\sqrt{{2}^{n}}\left\langle \left\langle P| {{\bf{D}}}_{{\boldsymbol{\omega }}}^{{\prime} }| 0\right\rangle \right\rangle$ returns an approximation $\tilde{g}$ for the cost function $\tilde{f}$ with error

24 $$\Delta (\tilde{f},\tilde{g})\le {(1-2{p}^{{\prime} })}^{\ell +1}\le {e}^{-2{p}^{{\prime} }\ell }$$

and runs in time at most *O*(*n*^2^*m*2^*ℓ*^).

The result relies on the fact that any Pauli channel will map a propagated Pauli operator to itself, up to a proportionality factor that can be at most 1. In other words, this means that each of the modified process modes ${{\mathcal{D}}}_{{\boldsymbol{\omega }}}^{{\prime} }$ will act similarly to the previously considered modes ${{\mathcal{D}}}_{{\boldsymbol{\omega }}}$ arising from the simplified error model, so that ${{{\bf{D}}}^{{\prime} }}_{{\boldsymbol{\omega }}}\left.\left\vert P\right\rangle \right\rangle \propto {{\bf{D}}}_{{\boldsymbol{\omega }}}\left.\left\vert P\right\rangle \right\rangle$. Therefore the proof and the bounds follow in the same way as for Theorem 1. The only modification to the algorithm is that to compute ${d}_{{\boldsymbol{\omega }}}^{{\prime} }$ one must also keep track of these proportionality factors along with the propagated Pauli.

For generality we have not assumed that the all noise coefficients of $\{{{\mathcal{M}}}_{i}\}$ are bigger than 0, thus it is difficult to improve upon the upper bound on the approximation error *Δ* since one can be in a situation where along the paths of weight ∣***ω***∣ = *ℓ* + 1 the proportionality factors might all be 1 when propagating the Pauli operator through each Pauli channel ${{\mathcal{M}}}_{i}$. In practical situations the Clifford gates will come with a depolarising component and the bound can be improved. For instance, let’s assume that in the decomposition of the *n* qubit Clifford operator ${{\mathcal{C}}}_{i}$ into primitive (single and two-qubit) gates each incurs a local single-qubit depolarising channel ${{\mathcal{N}}}_{dep}$ with error probability *η*. Then it follows we can find a tighter bound

25 $$\Delta (\tilde{f},\tilde{g})\le {(1-2{p}^{{\prime} })}^{\ell +1}{(1-\eta )}^{\ell +1}\le {e}^{-(2{p}^{{\prime} }+\eta )\ell }.$$

This comes from the fact that ${{\mathcal{N}}}_{dep}^{\dagger }(P)=(1-\eta )P$ if *P* ∈ {*X*, *Y*, *Z*} and ${{\mathcal{N}}}_{dep}^{\dagger }(I)=I$ along with the previous observation that for valid paths leading to non-zero coefficients, ${{\mathcal{D}}}_{\pm 1}$ are applied to qubit *q*_*i*_ whenever the propagated Pauli on qubit *q*_*i*_ is not *I* or *Z*. Therefore the noise from the Clifford part will contribute and at the very least contract by a factor of (1 − *η*) whenever we have a branching possibility to apply either ${{\mathcal{D}}}_{+1}$ or ${{\mathcal{D}}}_{-1}$, which are the only contributors to the total weight ∣***ω***∣. Note that this type of noise model has previously been considered in the context of noisy random circuit sampling^7^. The corresponding result for non-Pauli observables can be obtained similarly as before, giving the same additional factor in the runtime.

### Fixed (unparameterised) non-Clifford gates

The extension of lowesa to the case where non-Clifford unparameterised gates are present is straightforward. As was done in ref. ^47^, one may treat non-Clifford rotation gates as parameterised rotation gates that have their parameters fixed on at a later stage. A circuit with *t* fixed *z*-rotation gates and *m* parameterised *z*-rotation gates may be transformed into a circuit with *m* + *t* *z*-rotations for simulation purposes, obtaining a cost function *F*(***θ***, ***ϕ***). Then the intended cost function is obtained by fixing ***ϕ***. It follows that any statement on the simulation runtime still applies with the substitution *m* → *m* + *t*. However, getting an error bound with non-Clifford gates is more complicated, since we can no longer average over the expanded parameter space owing to the fixed gates. We can still make a weaker probabilistic statement, proven in Methods.

#### Theorem 5

Consider a variational circuit consisting of *m* uncorrelated noisy parameterised rotation gates, and *t* noisy rotation gates with fixed random angles independently and uniformly distributed in [0, 2*π*]^*t*^. The noise model is that of Theorem 4. Then for weight cutoff $\ell \in {\mathbb{N}}$, with probability ≥1 − *δ* the simulation error of lowesa (Algorithm 1) with modified process modes obeys

26 $$\Delta (\tilde{f}-\tilde{g})\le {e}^{-2{p}^{{\prime} }\ell }{\delta }^{-1/2}$$

and the Algorithm runs in time *O*(*n*^2^(*m* + *t*)2^*ℓ*^).

Theorem 5 implies that for a typical choice of the ***ϕ*** angles the error is still exponentially suppressed in *ℓ*. For fixed *δ*, one would choose $\ell \approx \frac{1}{2{p}^{{\prime} }}(\log {\epsilon }^{-1}+\frac{1}{2}\log {\delta }^{-1})$, giving a runtime which is only slightly worse than the one from the previous Theorems, for reasonable choices of *δ*.

### The case of correlated parameters

The main result has been derived assuming that the parameters controlling the rotation gates in the circuits are uncorrelated. One may therefore wonder whether it extends to correlated parameter circuits, which are ubiquitous in quantum machine learning^48^ as well as forming the basis of algorithms like the Quantum Approximate Optimisation Algorithm (QAOA)^32,49^ or the Hamiltonian Variational Ansatz (HVA) for chemistry problems^31,46^.

However, the argument used in the proof of Theorem 1 does not hold since with correlated angles the basis functions are no longer orthogonal over the correlated parameter space. For example, consider the following case where

27 $${\Phi }_{2}(\theta )={\cos }^{2}(\theta ),\,\,\,{\Phi }_{-2}(\theta )={\sin }^{2}(\theta )$$

28 $$\Rightarrow \frac{1}{2\pi }\int\,{\Phi }_{2}(\theta ){\Phi }_{-2}(\theta )d\theta =\frac{1}{8}\,\ne\, 0$$

Interestingly, we find that for correlated angles systems the simulation algorithm frequently returns a trivial result. Consider the following 1-qubit correlated parameter circuit

29 $${U}_{d}(\theta )={({R}_{x}(\theta ))}^{d}$$

It is simple to show that when *U*_*d*_(*θ*) is applied to the initial state $\left\vert 0\right\rangle$, then measuring the Pauli Z produces the cost function

30 $${f}_{d}(\theta )=\cos (d\theta )=\mathop{\sum }\limits_{i=0}^{\lfloor d/2\rfloor }{(-1)}^{i}\left(\begin{array}{c}d\\ 2i\end{array}\right)\,{\sin }^{2i}(\theta ){\cos }^{d-2i}(\theta )$$

whose terms are all of weight *d*. Therefore, any reconstruction with weight *ℓ* < *d* would trivially return $\tilde{g}=0$. This behaviour can be generalised to any circuit composed of *d* repeated, identical, and independently parameterised layers

31 $$U({\boldsymbol{\theta }})=\mathop{\prod }\limits_{i=1}^{d}V({{\boldsymbol{\theta }}}_{i})=\mathop{\prod }\limits_{i=1}^{d}\left(\mathop{\prod }\limits_{j=1}^{h}{e}^{-i{H}_{j}{\theta }_{ij}}\right),$$

where each layer is generated by the *same* *h* Hamiltonians. It can be observed that both QAOA and HVA ansatzes fit in the prescription. In this situation, for lowesa to produce a non-zero approximation function $\tilde{g}$, we show (see Methods) that the cutoff value *ℓ* has to be greater than the number of repeated layers.

#### Theorem 6

Given *U*(***θ***) as in Eq. (31) and a Pauli operator *P* that does not commute with at least one of the generators {*H*_*j*_}. If the cutoff *ℓ* < *d* then lowesa produces a trivial approximation $\tilde{g}=0$ of the noisy expectation value at any noise level.

This result implies that the complexity requirements lowesa will scale exponentially Ω(2^*d*^) with the number of layers. Correlating the angles further, for example by setting ***θ***_1_ = ***θ***_2_ = ⋯ = ***θ***_*p*_ does not affect the validity of the result. Improvements to the runtime may be possible if the number of valid paths can be reduced, for instance by leveraging symmetries in the circuit.

While the simulation algorithm may appear to fail for correlated angles since the output is constant for *ℓ* < *d*, in fact we have not considered that the simulation RMSE may still be small if the *noisy* cost function $\tilde{f}$ has a small variance, since (assuming *ℓ* < *d* and ${{\mathbb{E}}}_{{\boldsymbol{\theta }}}\tilde{f}=0$)

32 $${\Delta }^{2}(\tilde{f},\tilde{g})={\Delta }^{2}(\tilde{f},0)={{\mathbb{E}}}_{{\boldsymbol{\theta }}}{\tilde{f}}^{2}={{\rm{Var}}}_{{\boldsymbol{\theta }}}(\tilde{f}).$$

Indeed this is the case due to the phenomenon of noise-induced barren plateaus: for our model the cost function variance would decay with depth as $O({e}^{-pd/\ln 2})$ [^13^, Lemma 1]. Therefore it is still possible that the result in Theorem 1 may hold for correlated parameter VQAs too. For now, however, we are unable to conclusively demonstrate it, so this leaves room for a quantum advantage in QAOA and HVA, as well as in simulating time evolution on noisy quantum devices, as such tasks commonly involve repeated gate patterns.

## Discussion

In this work we introduce lowesa, an algorithm to approximately classically simulate the cost function of VQAs, given any observable that can be efficiently measured on a quantum computer. Crucially, the algorithm is constructive, in that it outputs a *function* of circuit parameters that approximates the entire noisy landscape rather the observable’s noisy expectation value at some fixed parameters. We show that for circuits with *independently* parameterised non-Clifford gates and efficiently measurable observables, our procedure gives a polynomial-time algorithm in both the number of qubits and depth, with an upper bound on the average error that decays exponentially with the physical error rate and a controllable cutoff parameter. The implication is that generic VQAs with independent parameters, fixed efficiently measurable observable and under constant physical gate error rate can be efficiently simulated classically.

We emphasize that the approximation error measure we employ is an average over the entire parameter space. The claim of efficient classical simulatability for estimating expectation values in the presence of noise should be understood for a *typical circuit* within a family of circuits with fixed structure (i.e. fixed Clifford unitaries on an arbitrary topology interleaved with arbitrary non-Clifford *z*-rotations) and measurement operator. For the case of a PQC with uncorrelated parameters, this corresponds to a typical parameter constellation. On the other hand, when the circuit family contains fixed, non-Clifford gates, then our results hold only probabilistically (Theorem 5). Thus, we do not claim the ability to efficiently simulate all noisy Clifford+*T* circuits. At the same time, the aforementioned result also indicates that, for given circuit parameters, the probability to get an approximation error larger than the target accuracy of our algorithm also decreases exponentially with the cutoff parameter *ℓ*. While the cutoff is tunable, the algorithm’s computational cost scales exponentially in *ℓ* in the worst case. This behaviour is similar to what was observed in ref. ^6^, which shows classical simulability of sampling from generic (random) noisy circuits except a zero-measure subset of (fixed, structured) circuits.

Note that all our results suffer from the same limitation: fixing the simulation error, the scaling of the cutoff with *p* implies a runtime of 2^Θ(1/*p*)^, meaning that we require 1/*p* ∈ *o*(*m*) otherwise we recover the noiseless scaling of the algorithm. Another perspective is that there is a *minimum noise probability threshold* of 1/*m* below which lowesa loses any claim of advantage. This is expected since, if one interprets *p* as the probability of error per rotation gate, then *mp* is the total expected number of errors, which we require to be ≫ 1 otherwise noise will not have a significant effect on the cost function. Thus the quantity *mp* may represent a crude measure for the capability of lowesa to simulate a noisy VQA.

Our work can also be placed within a broader range of research^6,7,15,16,20,50^, that aims to establish the extent to which noise in quantum computations hinders any potential quantum advantage. The works in refs. ^6,7^, which inspired our algorithm, are specific to the task of simulating random circuit sampling and thus rely on different assumptions on circuit structure and output state. Recent frameworks^51^ show, up to oracular access, that specific circuit structures can exhibit a noise-robust quantum advantage. Our results are consistent with this because of the intrinsically probabilistic nature of our claims. However, it has also been shown that finite noise can introduce an exponential separation between an algorithm for learning quantum states running on a fault-tolerant quantum computer vs a NISQ device^52^. Similarly, our results imply that, in the presence of sufficiently large levels of noise, a generic, wide range of VQAs become classically simulatable. This type of conclusion has been reached in ref. ^15^, where comparisons with classical algorithms lead to trade-offs between physical error rates and depth limitations on variational Hamiltonian optimisation algorithms. For tensor network approaches^20^, truncation error accuracy is impacted by connectivity and has only been empirically related to noise. In contrast, our approach gives a constructive classical algorithm to recover the entire cost function, with provable bounds on accuracy (for the circuit families considered) and does not assume a particular problem or architecture topology. We note that our results in their present form do not apply to variational algorithms that sample from the output state, such as QAOA or quantum generative modelling^53–56^. These may be avenues for future exploration.

Besides the implication for the complexity of noisy VQAs, lowesa may have a place as a useful simulation algorithm for the NISQ era. While for fixed physical error rate per gate our algorithm scales polynomially in the number of qubits and depth, the complexity grows exponentially with decreasing error rates, in the worst case. However, in practice, it may possible to have better scaling for realistic circuits, for instance if the cost function is dominated by low-weight terms. Our experiments (Fig. 3) provide some empirical evidence that this is the case, supplementing similar findings in ref. ^47^.

Being a simulation algorithm for the cost function, lowesa may be used to generate classical surrogates of VQAs^24,57^, allowing model training without the hybrid optimisation loop. Unlike to other algorithms for this task, it has the advantage of requiring no samples from the quantum computer. As such it may be more directly compared with tensor network methods^58,59^, with some important differences. Crucially, lowesa generates the entire cost function, and its efficiency does not directly depend on the entanglement of the state, attributes that may make it preferable to tensor networks in some situations, for instance when the entangling gates are not geometrically local. On the other hand, tensor networks can be used to approximate the circuit’s output state on which any expectation value or bitstring probability may be evaluated, which contrasts with lowesa’s dependency on the measurement operator and specificity to expectation values. In any case, by using lowesa for the noiseless setting one necessarily abandons the rigorous guarantees of accuracy established in this work. Nonetheless, there are promising signs that it may still be useful in this regime^60^. Without the requirement of accuracy guarantees one is also free to implement different heuristics for branch cutting, since our solution was tailored to the noise model. Similar algorithms with different branch cutting heuristics have shown promise^61–63^.

Finally, prior to publication an article was released that details a Fourier-based simulation algorithm with many similarities to the one presented here^61^. They employ an analogous Pauli back-propagation scheme with a path length cutoff, with the crucial difference that they consider a noiseless scenario, where the accuracy of the output is not guaranteed. However, the paper is an excellent alternative presentation of the underlying concept, and suggests that such low-weight algorithms may have a place in simulating exact variational quantum circuits. Future work may thus focus on establishing tighter bounds on the accuracy of low-weight simulation methods for VQAs of interest, including circuits with correlated parameters such as QAOA and HVA that are currently outside the reach of our results.

Classical simulation algorithms such as that presented here can not only serve as benchmarking tools for NISQ devices at larger scales but most importantly, they help establish a threshold where quantum computers, given sufficiently low physical error rates, produce results that are no longer reproducible with classical computing resources. From this perspective, they are essential tools to determine the full picture of resource requirements for practical quantum applications.

## Methods

### Proof of Theorem 1

Using Eq. (15) we can rewrite Eq. (18) as

33 $${\Delta }^{2}(\tilde{f},\tilde{g})=\frac{1}{| \Theta | }{\int}_{\Theta }{\left\vert \sum _{| {\boldsymbol{\omega }}| \ > \ \ell }{\Phi }_{{\boldsymbol{\omega }}}({\boldsymbol{\theta }}){d}_{{\boldsymbol{\omega }}}\right\vert }^{2}d{\boldsymbol{\theta }}$$

Then using the fact that the trigonometric monomials Φ_***ω***_ are orthogonal

34 $$\frac{1}{| \Theta | }{\int}_{\Theta }{\Phi }_{{\boldsymbol{\omega }}}({\boldsymbol{\theta }}){\Phi }_{{{\boldsymbol{\omega }}}^{{\prime} }}({\boldsymbol{\theta }})d{\boldsymbol{\theta }}={2}^{-| {\boldsymbol{\omega }}| }{\delta }_{{\boldsymbol{\omega }}{{\boldsymbol{\omega }}}^{{\prime} }}$$

and thus form a basis, we derive the appropriate Parseval’s theorem

35 $${\Delta }^{2}(\tilde{f},\tilde{g})=\sum _{| {\boldsymbol{\omega }}| > \ell }{2}^{-| {\boldsymbol{\omega }}| }| {d}_{{\boldsymbol{\omega }}}{| }^{2}$$

Now consider the Fourier coefficients *d*_***ω***_. Recall from “Strategy” that these are defined as $\sqrt{{2}^{n}}\left\langle \left\langle P| {{\bf{D}}}_{{\boldsymbol{\omega }}}| 0\right\rangle \right\rangle$, and that the process modes **D**_***ω***_ (and their adjoints) map one Pauli operator to another Pauli operator, scaled by products of the Pauli channel eigenvalues *q*_*X*/*Y*/*Z*_. Also note that both Pauli noise and Pauli rotation channels only act nontrivially on a Pauli operator *O* when *O*’s component on the qubits where the channel acts nontrivially is not identity. Since by our model (Eq. (4)) rotation gates are always followed by noise channels acting nontrivially on the same qubits, we can infer that whenever a rotation gate *R* acts nontrivially on a Pauli operator *O*, the output operator will also be rescaled by a noise-dependent coefficient. This coefficient will depend on how many components of *O* are nonidentity on the qubits where *R* acts nontrivially, but by the argument above there must be at least one such component. Therefore by our assumptions on the noise the operator is scaled by at most *q* = 1 − 2*p* < 1.

If we define the zero-noise coefficients ${d}_{{\boldsymbol{\omega }}}^{0}$ by setting *p*_*X*/*Y*/*Z*_ = 0, we see that $| {d}_{{\boldsymbol{\omega }}}| ={Q}_{{\boldsymbol{\omega }}}| {d}_{{\boldsymbol{\omega }}}^{0}|$, where 0 < *Q*_***ω***_ ≤ *q*^∣***ω***∣^. Hence we can write

36 $${\Delta }^{2}(\tilde{f},\tilde{g})=\sum _{| {\boldsymbol{\omega }}| > \ell }{Q}_{{\boldsymbol{\omega }}}^{2}{2}^{-| {\boldsymbol{\omega }}| }| {d}_{{\boldsymbol{\omega }}}^{0}{| }^{2}$$

37 $$\le {q}^{2(\ell +1)}\sum _{| {\boldsymbol{\omega }}| > \ell }{2}^{-| {\boldsymbol{\omega }}| }| {d}_{{\boldsymbol{\omega }}}^{0}{| }^{2}$$

38 $$\le {q}^{2(\ell +1)}\sum _{{\boldsymbol{\omega }}}{2}^{-| {\boldsymbol{\omega }}| }| {d}_{{\boldsymbol{\omega }}}^{0}{| }^{2}$$

Now we recognise that by Parseval’s theorem the summation relates to the noise-less cost function *f*(***θ***) as

39 $$\sum _{{\boldsymbol{\omega }}}{2}^{-| {\boldsymbol{\omega }}| }| {d}_{{\boldsymbol{\omega }}}^{0}{| }^{2}=\frac{1}{| \Theta | }{\int}_{\Theta }| f({\boldsymbol{\theta }}){| }^{2}d{\boldsymbol{\theta }}\le 1.$$

This implies $\Delta (\tilde{f},\tilde{g})\le {q}^{\ell +1}$, which gives a non-trivial bound whenever *q* < 1, which again is a reasonable assumption. To simplify the expression further, we use the identity $1-x\le {e}^{-x}\,\forall x\in {\mathbb{R}}$ and find *q*^*ℓ*+1^≤ *e*^−2*ℓ*^.

Finally, we compute the runtime of the algorithm to determine the approximation $\tilde{g}$. As outlined in the main text, we produce a binary tree-like data structure to keep track of the back-propagation of the target measurement Pauli operator *P* through the noisy circuit. This drastically improves the performance, as not all weight vectors produce valid paths that are non-zero. Thus, we only keep track of the paths leading to non-zero *d*_***ω***_.

We start with the target Pauli measurement *P* and we have *m* layers to propagate it through, beginning with ${{\mathcal{D}}}_{{\omega }_{m}}^{m\dagger }\,{\circ}\, {{\mathcal{C}}}_{m}^{\dagger }(P)$. As *C*_*m*_ is an *n*-qubit Clifford, *P* can be updated to another Pauli operator ${C}_{m}^{\dagger }(P)$ and this takes generically at most *O*(*n*^2^). Now ${{\mathcal{D}}}_{{\omega }_{m}}^{m}$ acts with Pauli generator *P*_*m*_. So if the propagated Pauli operator commutes with *P*_*m*_ then it forces ${{\mathcal{D}}}_{{\omega }_{m}}^{m}={{\mathcal{D}}}_{0}^{m}$. Otherwise if the propagated Pauli operator anticommutes with *P*_*m*_ then there are two possible choices ${{\mathcal{D}}}_{\pm 1}^{m}$ that do not give a zero process mode. In this case, we have two possible branches and we determine the propagated Pauli operators for each. Then each of these will act as input for the next layer where we repeat the same process. The update of the Pauli frame through each ${{\mathcal{D}}}_{{\omega }_{i}}^{i}$ takes *O*(1). Note that the Pauli frame is deterministically updated before any branches occur.

Therefore we produce a tree graph where nodes correspond to those propagated Pauli operators for which the next step requires two possibilities (i.e. apply ${D}_{-1}^{i}$ on one branch and ${D}_{+1}^{i}$ on the other). We also assign edges with values that track the number of *D*_0_’s that occurred between two consecutive nodes. As the weight of each paths is at most ∣***ω***∣ ≤ *ℓ*, then there are at most *ℓ* + 1 levels in the binary tree. Some of the branches will terminate sooner, but the maximal number of nodes in level *i* is 2^*i*^ for *i* ∈ [0, *ℓ*]. Note that updating the Pauli frame operator between any two consecutive nodes take *O*(*n*^2^ *k*) where *k* is the number of ${D}_{0}^{{\prime} }s$ applied in between. Therefore updating the layer *i* + 1 given all the Pauli operators in layer *i* takes $O({n}^{2}({k}_{1}+\ldots +{k}_{{2}^{i}}))$. However, since the number of ${D}_{0}^{{\prime} }s$ applied within any branch satisfies *k* ≤ *m*, then updating layer *i* + 1 given *i* takes at most *O*(*n*^2^2^*i*^*m*). Putting all together it means that propagating *P* through all valid paths takes at most $\mathop{\sum }\nolimits_{i = 0}^{\ell -1}O({n}^{2}{2}^{i}m)=O({n}^{2}{2}^{\ell }m)$.

Note that the scaling with *m* provides a coarse upper bound. If it is attained then that means there’s no branching in that specific tree and the complexity will in that case be independent of the cutoff too. The scaling with the number of qubits *n* depends on the details of the Clifford part of the circuit. In the worst case, when the Clifford layers are generic Cliffords, they can be represented as 2*n* × 2*n* symplectic matrices^64^, and therefore their application takes naïvely *O*(*n*^2^) time. Otherwise, if the Clifford layers consist of gates of maximum locality *k* and maximum depth *d*, the runtime is *O*(*k*^2^*nd*). In both cases the runtime is polynomial in *n*, as claimed.

### Proof of Theorem 3

By linearity

40 $$\tilde{f}({\boldsymbol{\theta }})-\tilde{g}({\boldsymbol{\theta }})=\sum _{i}{c}_{i}({\tilde{f}}_{i}({\boldsymbol{\theta }})-{\tilde{g}}_{i}({\boldsymbol{\theta }}))$$

where the subscript indicates that the observable *P*_*i*_ is measured. Once again the error is defined as

41 $${\Delta }^{2}={{\mathbb{E}}}_{{\boldsymbol{\theta }}}{(\tilde{f}({\boldsymbol{\theta }})-\tilde{g}({\boldsymbol{\theta }}))}^{2},$$

where again we assume the parameters are sampled from a uniform distribution.

Now we let ${e}_{i}({\boldsymbol{\theta }})={\tilde{f}}_{i}({\boldsymbol{\theta }})-{\tilde{g}}_{i}({\boldsymbol{\theta }})$. Expanding Eq. (41) using Eq. (40) and using Cauchy-Schwarz,

42 $${\Delta }^{2}=\sum _{i,j}{c}_{i}{c}_{j}\,{{\mathbb{E}}}_{{\boldsymbol{\theta }}}({e}_{i}{e}_{j})$$

43 $$\le \sum _{i,j}| {c}_{i}| | {c}_{j}| \,\sqrt{{{\mathbb{E}}}_{{\boldsymbol{\theta }}}({e}_{i}^{2}){{\mathbb{E}}}_{{\boldsymbol{\theta }}}({e}_{j}^{2})}$$

44 $$={\left(\sum _{i}| {c}_{i}| \sqrt{{{\mathbb{E}}}_{{\boldsymbol{\theta }}}({e}_{i}^{2})}\right)}^{2}$$

Now using Theorem 1 and letting the cutoff vary with *i* we get

45 $$\Delta \le \sum _{i}| {c}_{i}| {e}^{-2p{\ell }_{i}}$$

To take into account the simulation cost, we seek to minimise

46 $$\sum _{i}| {c}_{i}| {e}^{-2p{\ell }_{i}}+\lambda \sum _{i}{2}^{{\ell }_{i}}$$

with *λ* a Lagrange multiplier. The solution is

47 $${\ell }_{i}=\frac{1}{2p+\log 2}\log | {c}_{i}| +\log k$$

where *k* is a positive constant. The error is therefore

48 $$\Delta \le {k}^{-2p}\sum _{i}| {c}_{i}{| }^{\frac{\log 2}{\log 2+2p}}.$$

Alternatively, if we require Δ ≤ *ϵ*, the total simulation cost is

49 $$\sum _{i}{2}^{{\ell }_{i}}={k}^{\log 2}\sum _{i}| {c}_{i}{| }^{\frac{\log 2}{\log 2+2p}}$$

50 $$\le {\epsilon }^{-\frac{\log 2}{2p}}{\left(\sum _{i}| {c}_{i}{| }^{\frac{\log 2}{\log 2+2p}}\right)}^{1+\frac{\log 2}{2p}}$$

51 $$={({\epsilon }^{-1}\parallel c{\parallel }_{r})}^{\frac{\log 2}{2p}}$$

with $r=\frac{\log 2}{\log 2+2p}$, which ≈ 1 when $2p\,\ll\, \log 2$. Therefore the runtime is in

52 $$O({({\epsilon }^{-1}\parallel c{\parallel }_{r})}^{\frac{\log 2}{2p}}{n}^{2}m).$$

### Proof of Theorem 4

The main difference from the proof of Theorem 1 is that the *n*-qubit unitaries ${{\mathcal{C}}}_{i}$ are noisy and are replaced by ${{\mathcal{M}}}_{i}\,{\circ}\, {{\mathcal{C}}}_{i}$. However, since ${{\mathcal{M}}}_{i}$ are Pauli channels, then its adjoint acts on any Pauli operator as ${{{\bf{M}}}_{{\bf{i}}}}^{T}\left.\left\vert P\right\rangle \right\rangle \propto \left.\left\vert P\right\rangle \right\rangle$, where the proportionality factor is determined by the eigenvalues of ${{\mathcal{M}}}_{i}$. These are assumed to be accessible, e.g. from previous benchmarking experiments. Therefore, the total number of valid, non-zero process modes is also 2^*ℓ*^ and there are *m* Pauli channels ${{\mathcal{M}}}_{i}$ so computing the proportionality factor takes at most *O*(*m* 2^*ℓ*^), which means it does not affect the overall complexity in determining the Fourier coefficients ${d}_{{\boldsymbol{\omega }}}^{{\prime} }=\sqrt{{2}^{n}}\left\langle \left\langle P| {{\bf{D}}}_{{\boldsymbol{\omega }}}^{{\prime} }| 0\right\rangle \right\rangle$ with ∣***ω***∣ ≤ *ℓ* which can be computed, as previously, in *O*(*n*^2^*m*2^*ℓ*^).

It remains to show that the average approximation error $\Delta (\tilde{f},\tilde{g})$ still decays exponentially with the cutoff parameter *l*. Like before, the noisy cost function is given by

53 $$\tilde{f}({\boldsymbol{\theta }})=\sum _{{\boldsymbol{\omega }}}{d}_{{\boldsymbol{\omega }}}^{{\prime} }{\Phi }_{{\boldsymbol{\omega }}}({\boldsymbol{\theta }})$$

where ${d}_{{\boldsymbol{\omega }}}^{{\prime} }={Q}^{{\prime} }({\boldsymbol{\omega }}){d}_{{\boldsymbol{\omega }}}^{0}$, ${Q}^{{\prime} }({\boldsymbol{\omega }})\le {q}^{| {\boldsymbol{\omega }}| }$ if the noise ${\mathcal{N}}$ on the parameterised gates ${{\mathcal{R}}}_{i}({\theta }_{i})$ is a fixed, space- and time-independent Pauli channel with eigenvalues (*q*_*X*_, *q*_*Y*_, *q*_*Z*_) and $q:= \mathop{\max}\nolimits_{\sigma }\{{q}_{\sigma }\}$. More generally however, if ${\mathcal{N}}$ carries a space- and time-dependency with possibly different eigenvalues $({q}_{X}^{ij},{q}_{Y}^{ij},{q}_{Z}^{ij})$ for each of the parameterised gates ${{\mathcal{R}}}_{i}({\theta }_{i})$ and qubit *j*, then we have ${Q}^{{\prime} }({\boldsymbol{\omega }})\le {\prod }_{i}{({q}^{i})}^{| {\omega }_{i}| }$, where ${q}^{i}:= \mathop{\max}\nolimits_{j\sigma }\{{q}_{\sigma }^{ij}\}$. Note that in this situation, the process modes for each site ${{\mathcal{D}}}_{{\omega }_{i}}^{i}$ will have the same form as in the previous analysis but with different parameters that depend on the location.

Finally, orthogonality of the trigonometric functions Φ_***ω***_(**θ**) ensures we get

54 $${\Delta }^{2}(\tilde{f},\tilde{g})=\sum _{| {\boldsymbol{\omega }}| > \ell }{Q}^{{\prime} 2}({\boldsymbol{\omega }}){2}^{-| {\boldsymbol{\omega }}| }| {d}_{{\boldsymbol{\omega }}}^{0}{| }^{2}$$

55 $$\le {(\mathop{\max }\limits_{| {\boldsymbol{\omega }}| \ > \ \ell }{Q}^{{\prime} }({\boldsymbol{\omega }}))}^{2}\sum _{{\boldsymbol{\omega }}}{2}^{-| {\boldsymbol{\omega }}| }| {d}_{{\boldsymbol{\omega }}}^{0}{| }^{2}$$

The term within the brackets is the largest (*ℓ* + 1) product of the *q*^*i*^’s. This can be given the trivial upper bound $1-2{p}^{{\prime} }$ by defining ${p}^{{\prime} }:= \mathop{\min}\limits_{ij\sigma }\{{p}_{\sigma }^{ij}\}$ and ${p}_{Z}^{{\prime} }:= \mathop{\min }\nolimits_{i}\{{p}_{Z}^{i}\}$. In this case we must have ${p}^{{\prime} }\, >\, 0$. Finally, the sum ${\sum }_{{\boldsymbol{\omega }}}{2}^{-| {\boldsymbol{\omega }}| }| {d}_{{\boldsymbol{\omega }}}^{0}{| }^{2}$ can be bounded by 1 as explained in the proof of Theorem 1.

### Proof of Theorem 5

We can use the trick of considering the random *t* angles to be variables on a space Φ = [0, 2*π*]^*t*^, and replicate the proofs of Theorems 1 and 4 on the expanded parameter space Θ ⊗ Φ = [0, 2*π*]^*m*+*t*^. This gives the bound

56 $${{\mathbb{E}}}_{{\boldsymbol{\phi }}}{\Delta }^{2}(\tilde{f},\tilde{g})=\frac{1}{| \Theta | | \Phi | }{\int}_{\Phi }{\int}_{\Theta }| \tilde{f}({\boldsymbol{\theta }},{\boldsymbol{\phi }})-\tilde{g}({\boldsymbol{\theta }},{\boldsymbol{\phi }}){| }^{2}d\theta d\phi$$

57 $$\begin{array}{l}\le {e}^{-4{p}^{{\prime} }\ell }\end{array}$$

Now we can use Markov’s inequality on the random variable $\Delta (\tilde{f},\tilde{g})$ (as it now depends on the random ***ϕ***) and obtain the intended result. The running time is bounded by *O*(*n*^2^(*m* + *t*)2^*l*^) since the number of rotation gates is now *m* + *t*.

### Proof of Theorem 6

We state the following Lemma:

#### Lemma 7

Consider a circuit in the form of Eq. (31) and its expansion in process modes. Then given a Pauli operator *P*, either i) it commutes with all generators {*H*_*j*_}, or ii) the process modes that do not annihilate *P* all have weight ∣***ω***∣ ≥ *d*.

#### Proof

First note that, given a Pauli operator *P*_*i*_ and a Hermitian *H* with [*P*_*i*_, *H*] ≠ 0, then the corresponding unitary acts nontrivially on *P*, more precisely

58 $${e}^{-iH\theta }{P}_{i}{e}^{iH\theta }=\sum _{j}{c}_{j}{P}_{j}$$

with *c*_*j*_ ≠ 0 for at least one *j* ≠ *i*. In that case it is easy to show that [*P*_*j*_, *H*] ≠ 0 for all such a *P*_*j*_.

Therefore we see that if at layer *i* the unitary ${e}^{-i{H}_{j}{\theta }_{ij}}$ acts nontrivially on a Pauli *P*, even if all its products commute with all subsequent unitaries, then they cannot also commute with the next unitary generated by *H*_*j*_. This means that either *P* commutes with all generators, or at least one unitary must act nontrivially per layer. In the latter case, it follows that the weight of any process mode that does not annihilate *P* must necessarily be greater than or equal to the total number of layers *d*. □

The theorem follows simply by observing that if *ℓ* < *d*, Algorithm 1 must return $\tilde{g}({\boldsymbol{\theta }})=0$ since all process modes annihilate *P*. The result holds equally if noise is present as noise simply adds up to a constant for each mode in the expansion.

The following corollary also follows from Lemma 7, which we report as it may be of independent interest:

#### Corollary 8

Consider a unitary *U* in the form of Eq. (31). Then for any Hermitian *O*, the resulting cost function *f*(***θ***) consists only of terms of weight ≥*d* and constant terms.

This follows simply from expanding *O* in Pauli operators and applying the Lemma.

### Fourier decomposition under dephasing channel

In this example we illustrate the contraction of Fourier coefficients with the Hamming weight of the frequency vector under a dephasing noise model. While we had to use the trigonometric basis instead of complex exponentials in order to obtain the efficient classical simulation, this example gives a cleaner intuition behind the low-weight approximation.

Here, we use a simplified time-independent noise model

59 $${\tilde{{\mathcal{U}}}}_{{\boldsymbol{\theta }}}=\left({\bigcirc}_{i}\,{{\mathcal{C}}}_{i}\,{\circ}\, {{\mathcal{N}}}_{phase}(p)\,{\circ}\, {{\mathcal{R}}}_{z}^{({q}_{i})}({\theta }_{i})\right)\,{\circ}\, {{\mathcal{C}}}_{0},$$

where each parameterised single qubit rotation is affected by a dephasing channel.

In ref. ^21^ it was shown that a rotation gate affected by dephasing noise can be decomposed as a linear combination of channels, each carrying an oscillatory term in *θ* and a noise term in *p*. This was termed the *process mode decomposition* as it decomposes a channel (aka a quantum process) into Fourier modes^26^. For the specific case of the *z*-rotation channel affected by dephasing, the decomposition is

60 $${{\mathcal{N}}}_{phase}(p)\,{\circ}\, {{\mathcal{R}}}_{z}(\theta )={{\mathcal{C}}}_{0}+(1-2p){e}^{i\theta }{{\mathcal{C}}}_{1}+(1-2p){e}^{-i\theta }{{\mathcal{C}}}_{-1}$$

where the process modes ${{\mathcal{C}}}_{i}$ are linear combinations of Clifford unitary channels. Note that such decompositions are generally not unique, and indeed we will consider a different decomposition shortly.

Therefore, it follows that under phase noise, the noisy PQC defined in Eq. (59) can be given a process mode decomposition, where each mode is a compositions of Clifford channels and single-qubit modes ${{\mathcal{C}}}_{i}$ labelled by a frequency vector ***ω*** ∈ [0, ±1]^*m*^:

61 $${{\mathcal{C}}}_{{\boldsymbol{\omega }}}:= \left({\bigcirc}_{i}\,{{\mathcal{U}}}_{i}\,{\circ}\, {{\mathcal{C}}}_{{\omega }_{i}}\right)\,{\circ}\, {{\mathcal{U}}}_{0}$$

The decomposition is

62 $${\tilde{{\mathcal{U}}}}_{{\boldsymbol{\theta }}}=\sum _{{\boldsymbol{\omega }}\in {\{0,\pm 1\}}^{m}}{(1-2p)}^{| {\boldsymbol{\omega }}| }{e}^{i{\boldsymbol{\omega }}\cdot {\boldsymbol{\theta }}}{{\mathcal{C}}}_{{\boldsymbol{\omega }}}$$

Each mode is weighted by a noise term (1−2*p*)^∣***ω***∣^, where ∣***ω***∣ = ∑_*i*_∣*ω*_*i*_∣ is the Hamming weight of the mode. Now by linearity, the cost function can be written as

63 $$\tilde{f}({\boldsymbol{\theta }})=\sum _{{\boldsymbol{\omega }}\in {\{0,\pm 1\}}^{m}}{(1-2p)}^{| {\boldsymbol{\omega }}| }{c}_{{\boldsymbol{\omega }}}{e}^{i{\boldsymbol{\omega }}\cdot {\boldsymbol{\theta }}}$$

where ${c}_{{\boldsymbol{\omega }}}={\rm{tr}}(P{{\mathcal{C}}}_{{\boldsymbol{\omega }}}({\rho }_{0}))$ are the noiseless Fourier coefficients of the decomposition. One can see that with phase noise (*p* > 0) the Fourier coefficients are contracted as found in ref. ^21^.

## Data Availability

Data generated and analyzed during the current study are available from the corresponding author upon reasonable request.

## Code availability

The code used for the current study is available from the corresponding author upon reasonable request. A refined version of the code will soon be available via the PauliPropagation.jl package.
